# Multi-omic regulatory networks capture downstream effects of kinase inhibition in *Mycobacterium tuberculosis*

**DOI:** 10.1038/s41540-020-00164-4

**Published:** 2021-01-29

**Authors:** Albert T. Young, Xavier Carette, Michaela Helmel, Hanno Steen, Robert N. Husson, John Quackenbush, John Platig

**Affiliations:** 1 School of Medicine, University of California, San Francisco, USA; 2 Division of Infectious Diseases, Boston Children’s Hospital, Boston, USA; 3 Harvard Medical School, Boston, USA; 4 Department of Pathology, Boston Children’s Hospital, Boston, USA; 5 Department of Biostatistics, Harvard T.H. Chan School of Public Health, Boston, USA; 6 Channing Division of Network Medicine, Brigham and Women’s Hospital, Boston, USA

**Keywords:** Regulatory networks, Microbiology

## Abstract

The ability of *Mycobacterium tuberculosis (Mtb)* to adapt to diverse stresses in its host environment is crucial for pathogenesis. Two essential *Mtb* serine/threonine protein kinases, PknA and PknB, regulate cell growth in response to environmental stimuli, but little is known about their downstream effects. By combining RNA-Seq data, following treatment with either an inhibitor of both PknA and PknB or an inactive control, with publicly available ChIP-Seq and protein–protein interaction data for transcription factors, we show that the *Mtb* transcription factor (TF) regulatory network propagates the effects of kinase inhibition and leads to widespread changes in regulatory programs involved in cell wall integrity, stress response, and energy production, among others. We also observe that changes in TF regulatory activity correlate with kinase-specific phosphorylation of those TFs. In addition to characterizing the downstream regulatory effects of PknA/PknB inhibition, this demonstrates the need for regulatory network approaches that can incorporate signal-driven transcription factor modifications.

## Introduction

*Mycobacterium tuberculosis* (*Mtb*) remains one of the world’s deadliest pathogens, with 10 million people falling ill with tuberculosis (TB) and 1.5 million people dying from TB in 2018^1^. The emergence of multidrug-resistant and extensively drug-resistant *Mtb* strains threatens to undermine global TB control efforts.

Much of *Mtb*’s success as a pathogen can be attributed to its ability to adapt to diverse environmental stresses encountered during the course of chronic infection^2^. Protein kinases anchored on the mycobacterial cytoplasmic membrane are critical for responding to environmental stimuli and transducing signals to various cellular processes^3^. The essential *Mtb* serine/threonine protein kinases (STPKs) PknA and PknB are excellent targets to characterize in the context of future drug development, as they regulate several processes required for cell growth and division, including the biosynthesis of essential components of the cell envelope (peptidoglycan, mycolic acids, and other cell wall lipids and carbohydrates)^4^. For example, cells in which *PknA* or *PknB* gene expression was inhibited displayed an abnormal shape, indicating the two kinases are key regulators of cell division and cell shape in *Mtb*^5^. However, our understanding of the downstream transcriptional pathways by which PknA and PknB regulate these and other cellular processes is limited, and the basis of their essentiality is unknown.

**Fig. 1:**
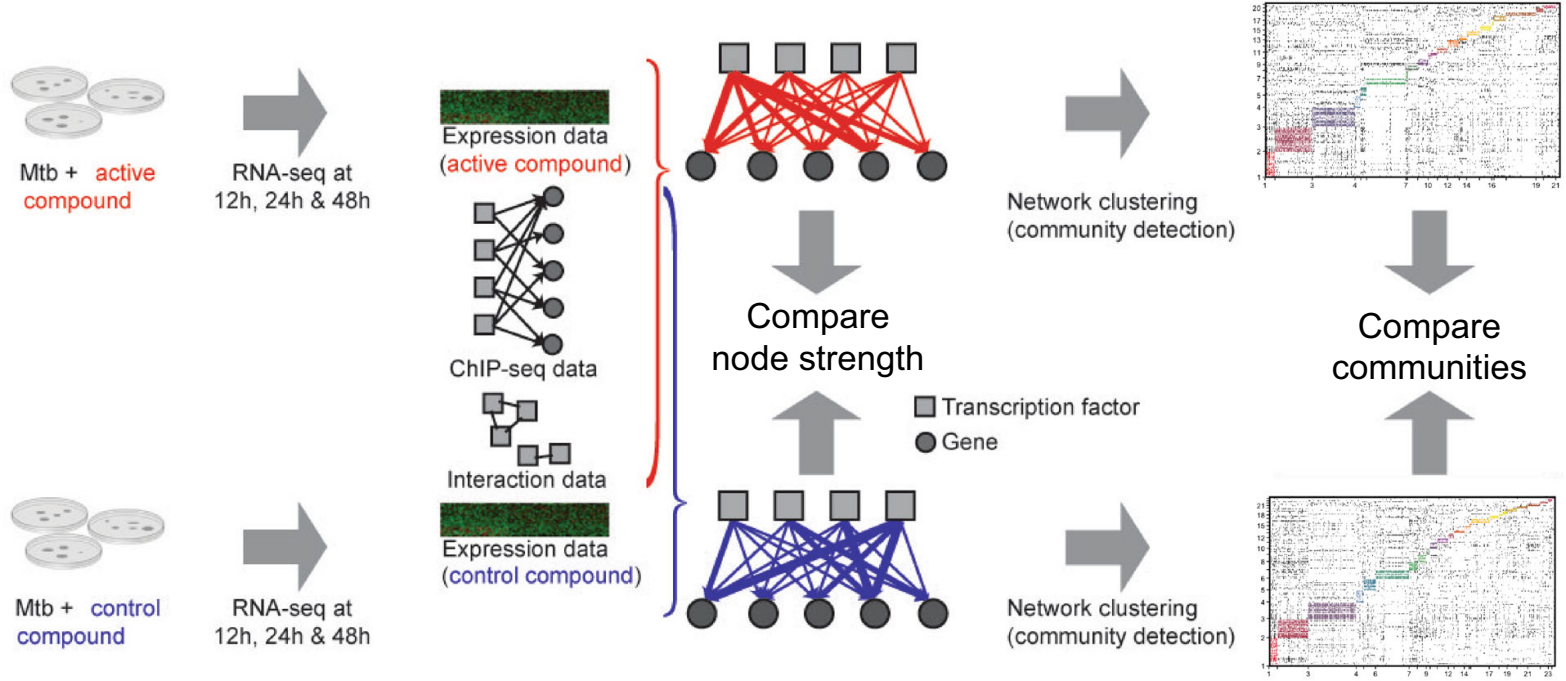
Summary of the experimental design and network comparisons. Using a message passing framework^9^ (see “Methods”) we integrated ChIP-seq and transcription factor protein–protein interaction (PPI) data with condition-specific gene expression data to build kinase inhibitor and control gene regulatory networks. Network edges connect TFs to genes and are weighted to reflect the confidence (in *z*-score units) of a regulatory relationship based on the concordance between omics data types. The TF node strength is defined as the sum of all weights for edges emanating from the TF, and gene node strength is the sum of all weights for edges terminating at the gene. The inhibitor and control networks were clustered into groups containing both TFs and genes using bipartite community detection^25,29^ as detailed in the “Methods”.

To measure the downstream effects of PknA/PknB signaling, we used a potent small molecule inhibitor of both PknA and PknB along with an inactive negative control, then collected multiple omics data types as detailed in ref. ^6^. To follow the propagation of this signaling perturbation, we sought to identify differences in transcription factor (TF) regulation in the inhibitor- and control-treated gene expression programs by reconstructing TF regulatory networks in the active (inhibitor) and control conditions. While condition-specific binding information for all TFs was not available, we combined recently generated ChIP-seq data for 143 TFs (as defined in ref. ^7^) in *Mtb* and protein–protein interaction data^8^ with RNA-Seq data from inhibitor- or control-treated samples using the TF regulatory network reconstruction algorithm PANDA^9^ (Fig. 1).

By comparing network topologies of these inhibitor and control networks, we first identify TFs that change their regulation in response to PknA/PknB inhibition as measured by change in TF node strength (sum of outbound edge weights). We also show that change in TF node strength is correlated with change in phosphorylation status of the TF after PknA/PknB inhibition, suggesting that our network approach is modeling the downstream effects of the kinase inhibition. Second, we show that genes that are differentially regulated as measured by their change in gene node strength (sum of inbound edge weights) are enriched for multiple functions, including mycobactin synthesis, with additional validation that mycobactin levels are indeed changed upon PknA/PknB inhibition^6^. Third, we demonstrate that network “communities” (modules) in the inhibitor-treated network show condition-specific functional enrichment, which are validated by follow-up experiments.

## Results

We created separate inhibitor- and control-specific TF regulatory networks by combining inhibitor- and control-treated gene expression data, respectively, with publicly available TF binding and protein–protein interaction data using PANDA^9^ (see “Methods” for details). For each network PANDA outputs a fully connected bipartite graph with 568,282 weighted edges between 143 TFs and 3974 genes. The weight of each edge between a TF and gene can be interpreted as a *z*-score representing the confidence of a regulatory relationship. While we found that many edge weights were highly similar in both conditions (Supplementary Fig. 1), our top 100 edges with the greatest change in edge weight connected 10 TFs and 67 genes, suggesting that multiple TFs and genes were responding to the kinase perturbation. To investigate this more systematically, we focused first on the network changes around each TF.

### Comparing networks uncovers PknA/PknB-specific targeting patterns of TFs

To identify TFs that appear to change their regulation in response to the kinase inhibitor, we used the sum of all outbound edge weights for a TF (“TF node strength”). We define the difference in TF node strength between kinase inhibitor- and control-treated networks as “differential targeting.” We consider “PknA/PknB-specific TFs” to be TFs with statistically significant differential targeting (see “Methods” for details).

**Fig. 2:**
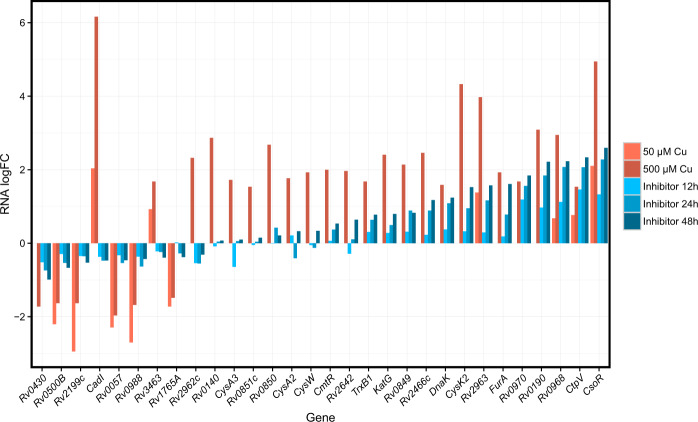
The transcriptional response of the CsoR regulon to PknA/PknB inhibition mirrors that of copper exposure. Log2 fold changes in RNA for genes in the CsoR regulon after treatment with the PknA/PknB inhibitor are shown in blue, changes in RNA after treatment with copper are shown in red (copper data from^15^). The copper-sensitive operon repressor (CsoR) showed the greatest change in phosphorylation and increase in TF node strength upon PknA/PknB inhibition (Fig. 3).

These PknA/PknB-specific TFs (Table 1 and Supplementary Data 1) are involved in processes we expect to be differentially regulated in the context of PknA/PknB inhibition. Rv0081, the most differentially targeting TF, is a regulatory hub in the context of hypoxia, a condition that induces a stress response with similarities to PknA/PknB inhibition^10^. Other top differentially targeting TFs include the response regulator TrcR, which activates its own coding gene expression and represses *Rv1057*, a *β*-propeller protein gene whose expression is also mediated by SigE^11^. Lsr2 is a global transcriptional regulator that may be responsible for many cell wall functions and is required for adaptation to changing oxygen levels^12,13^. CsoR, the TF with the greatest increase in targeting in kinase inhibitor-treated cells, is a copper sensing transcriptional regulator that may promote *Mtb* survival by mediating a response to copper toxicity^14^. The transcriptional response of the CsoR regulon to PknA/PknB inhibition mirrors that of copper exposure^15^ (Fig. 2). KstR, a transcriptional repressor, controls a number of genes involved in cholesterol and fatty acid catabolism^16^. Thus, each of the top 10 PknA/PknB-specific TFs with known function is involved in either signal transduction, cell wall function, or lipid metabolism-processes, which PknA and PknB regulate. Given this, we propose Rv0678, Rv0324, Rv0465c, Rv1985c, and Rv0023, which have unknown functions, to be candidate downstream regulators most affected by PknA/PknB inhibition as determined by change in TF node strength.

**Table 1:** Top ten transcription factors ranked by absolute change in node strength between inhibitor and control networks. *P*-values are calculated from permutations (see “Methods”).

RV	Node strength diff.	*P*-value	Genes	Product
Rv0081	−160.8	0.001		transcriptional regulator, ArsR family
Rv1033c	−123.6	0.001	*TrcR*	DNA-binding response regulator TrcR
Rv0678	−118.1	0.001		Conserved protein
Rv0324	−108.2	0.001		Transcriptional regulator, ArsR family
Rv3597c	−106.9	0.001	*Lsr2*	Histone protein Lsr2
Rv0967	97.2	0.021	*CsoR*	Copper-sensitive operon repressor CsoR
Rv0465c	−89.0	0.005		Transcriptional regulator, XRE family
Rv1985c	−84.2	0.001		HTH-type transcriptional regulator
Rv3574	−82.4	0.005	*KstR*	Transcriptional regulator kstR (Rv3574), TetR family
Rv0023	−77.5	0.002		Transcriptional regulatory protein

### Change in TF node strength is associated with change in TF phosphorylation

**Fig. 3:**
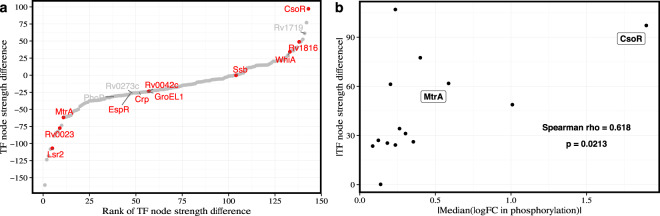
Change in transcription factor phosphorylation is correlated with change in node strength. **a** The difference in node strength (*y*-axis) and rank of difference in node strength (*x*-axis) are shown for the 143 transcription factors included in the regulatory network model. Of those, 14 TFs had detected phosphopeptides and 11 were differentially phosphorylated (adj. *P* < 0.05, shown in red). **b** The magnitude of change in TF node strength between active and control kinase inhibitor networks correlates with change in phosphorylation (Spearman correlation, *ρ*_*s*_ = 0.618 and *P* = 0.0213). The median log2 fold change was used when multiple phosphopeptides were detected for the same protein.

To test whether the signaling perturbation of PknA/PknB inhibition was detected in our networks, we used phosphoproteomic data collected from samples treated with the PknA/PknB inhibitor or control and calculated *log*_2_ fold change values to determine the phosphorylation status of the TFs in our networks. Peptides for 14 TFs were detected, 11 of which were differentially phosphorylated at an adjusted *P*-value < 0.05. We then compared the change in node strength between the inhibitor and control networks for each TF to the change in phosphorylation (Fig. 3) and find that these are significantly correlated (Spearman’s rho of 0.618, *P* = 0.0213). MtrA is one such TF. It has the 13th (out of 143) largest change in node strength between the inhibitor and control networks. MtrA is also ranked third in differential phosphorylation among the 14 TFs measured (Fig. 3). We also recently demonstrated that phosphorylation of MtrA inhibits DNA binding to the *FbpB* promoter^6^.

This discovery suggests that changes in transcriptional regulation in response to PknA/PknB inhibition can be partly attributed to differential phosphorylation of TFs. In particular, CsoR, the most positively differentially targeting TF, is significantly differentially phosphorylated with a log2 fold change of −1.90. That the majority (8/11) of differentially phosphorylated TFs in the network have decreased node strength in the inhibitor versus control network likely reflects the disruption of gene regulatory programs in response to PknA/PknB inhibition (that is, expression of genes in TF regulons are less well correlated).

### Change in gene node strength reveals differentially regulated functional categories

Among the genes that are “differentially targeted,” which we define as the difference in gene node strength (sum of all inbound edge weights) between control and inhibitor networks (Table 2 and Supplementary Data 2), we discovered many genes with interesting functions that may play important roles in responding to PknA/PknB inhibition. VapB30 and VapB40 are antitoxins in the VapBC (Virulence-associated protein) family of toxin-antitoxin systems that regulate translation in response to diverse environmental stresses^17^, and both *VapB30* and *VapB40* have increased gene expression and increased node strength in kinase inhibitor-treated cells. CysD is involved in sulfur metabolism, which may be important in the virulence, antibiotic resistance, and antioxidant defense mechanisms of *Mtb*^18^. ArgC, N-acetyl-gamma-glutamyl-phosphate reductase, is involved in arginine biosynthesis, an essential metabolic function for cellular growth and a pathway that is required for virulence^19,20^. Overall, we find a positive relationship between how differentially targeted a gene is and its differential expression (Supplementary Fig. 2).

**Table 2:** Top ten genes ranked by absolute change in node strength between inhibitor and control networks. *P*-values are calculated from permutations (see “Methods”).

RV	Node strength diff.	*P*-value	Genes	Product
Rv2913c	64.6	0.001		N-acyl-d-glutamate amidohydrolase
Rv0623	63.2	0.001	*VapB30*	Possible antitoxin VapB30
Rv3182	47.5	0.001		Conserved hypothetical protein
Rv1285	46.8	0.001	*CysD*	Sulfate adenylyltransferase subunit 2 (EC 2.7.7.4)
Rv1652	41.2	0.001	*ArgC*	N-acetyl-gamma-glutamyl-phosphate reductase (EC 1.2.1.38)
Rv2282c	−37.9	0.001	*CysB*	Cys regulon transcriptional activator CysB
Rv3453	37.4	0.001		Probable conserved transmembrane protein
Rv1219c	37.3	0.001		Transcriptional regulator, TetR family
Rv2595	36.2	0.001	*VapB40*	Possible antitoxin VapB40
Rv1693	−35.1	0.001		Conserved hypothetical protein

Using gene set enrichment analysis (GSEA) for difference in gene node strength, we identified PknA/PknB-specific functional categories (Supplementary Data 3). “Mycobactin biosynthesis,” “phosphopantetheine binding,” and “ESX-1 LOCUS” show increased targeting in the inhibitor condition, and “Oxidative phosphorylation,” “quinone binding,” and “NADH dehydrogenase” show decreased targeting. Mycobactins are essential for iron acquisition within the host environment^21^, and in related work (Figure 4 of ref. ^6^), we found that mycobactin levels were increased 48 hours after inhibition of PknA/PknB. “Phosphopantetheine binding” consists of 15 genes, including polyketide synthases crucial for fatty acid synthesis^22^ and enzymes involved in mycobactin synthesis^23^. The ESX-1 system is a specialized secretion system required for virulence^24^. Oxidative phosphorylation, quinone binding, and NADH dehydrogenase genes are less targeted after PknA/PknB inhibition, supporting their role in mediating a compensatory biological response in the form of lowered energy expenditure and growth arrest.

### Network clustering reveals condition-specific communities with different biological functions

While differential targeting analysis gives insight at the whole-network level, we aimed to understand biological organization at a more modular level. As PANDA models groups of TFs regulating groups of genes, we naturally partitioned the nodes from each network into communities. To facilitate comparison of the network clusters, we considered only the set of edges with positive edge weights (*z*-score > 0) in both conditions. This resulted in a Giant Connected Component (GCC) containing 67,740 edges between 143 TFs and 3971 genes. Genes that are also TFs were included as separate nodes in the network. As a network diagnostic, we plotted the distributions of TF node strength and gene node strength for the thresholded PANDA networks (Supplementary Fig. 3).

Next, we used CONDOR (COmplex Network Description Of Regulators), an R package for bipartite network analysis^25^, to detect communities independently in each PANDA network. CONDOR maximizes the modularity, a score that can be interpreted as an enrichment for links within communities minus an expected enrichment given the network degree distribution.

This analysis identified 21 communities in the inhibitor network and 24 communities in the control network, with similar modularities of 0.495 and 0.504, respectively, and similar membership (Supplementary Fig. 4 and Supplementary Fig. 5). By testing each community as a whole for functional enrichment (see “Methods”), we found 4 of the 21 communities in the inhibitor network and 3 of the 24 communities in the control network to be functionally enriched (FDR < 0.05; overlap > 4) in one or more functional categories (Supplementary Data 4 and Supplementary Data 5). This functional enrichment is robust to the choice of edge weight threshold (Supplementary Fig. 6). The differences in the enrichment identified between conditions are relevant in the context of PknA/PknB inhibition. For example, response to stress is enriched in a community in the inhibitor but not the control network; 11 of 26 genes in the functional category are assigned to community 2 in the inhibitor network, suggesting that these genes are cooperatively regulated in response to PknA/PknB inhibition. In addition to the activation of stress response, the results suggest that energy metabolism and ATP production regulation change in response to PknA/PknB inhibition.

**Fig. 4:**
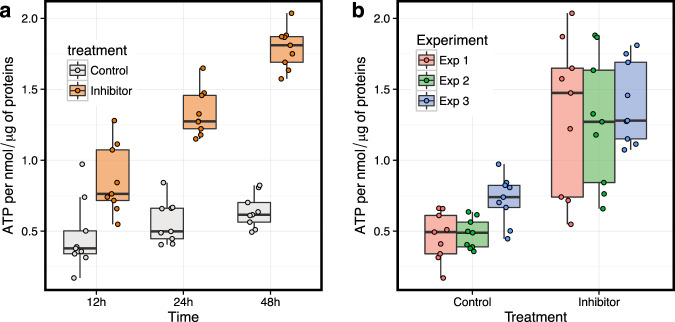
ATP production increases after inhibition of PknA/PknB. **a** ATP levels are higher for inhibitor-treated samples compared to controls at all time points and **b** across three experiments (*P* = 3.213 × 10^−10^; *t* = 8.597; df = 35.696; 95% CI [0.59, 0.954]; for two-sided *t*-test comparing inhibitor vs. control). Samples were normalized to the amount of residual protein (see “Methods” for details). Each boxplot displays the median (middle line), the first and third quartiles (lower and upper hinges) and the most extreme values no further than 1.5* the interquartile range from the hinge (upper and lower whiskers).

To validate the network finding that ATP metabolism is disrupted by inhibition of PknA/PknB, we quantified the level of ATP in both the control and inhibitor-treated cells at 12, 24, and 48 hours. After normalizing based on residual protein quantity (see “Methods”), we observed higher levels of ATP at all time points in the inhibitor-treated samples compared to the control-treated samples (Fig. 4).

## Discussion

As multi-omic data acquisition becomes increasingly commonplace, researchers interested in the drivers of complex phenotypes will face a new intellectual challenge: given a wealth of omics data, how can one identify relevant regulatory relationships amidst an abundance of correlations and partial correlations within and across data types? One solution to this challenge is to build regulatory networks that move beyond simple correlations by creating models that are consistent with known mechanisms of gene regulation. Here, we applied one such algorithm, PANDA, to estimate the TF regulatory network response to an antimicrobial compound that inhibits two key signaling molecules important for cell wall function and stress response in *Mycobacterium tuberculosis*, PknA and PknB.

By comparing the regulatory networks from samples treated with either the PknA/PknB inhibitor or an inactive control compound, we identified treatment-specific network changes and provide validation for multiple network-generated hypotheses. This includes the observation that changes in TF node strength correlate with changes in TF phosphorylation, suggesting that the PANDA regulatory networks successfully capture the relevant downstream effects of the perturbation. We identified “differentially targeting” PknA/PknB-specific TFs (based on change in TF node strength) that affect diverse biological functions associated with signal transduction, cell wall function, and lipid metabolism. Additionally, we identified “differentially targeted” PknA/PknB-specific genes (based on change in gene node strength) that our models predict have a condition-specific pattern of regulation. In the case of mycobactin synthesis, these network changes are corroborated by changes in mycobactin lipid levels.

Networks are known to exhibit complex substructure, and we found the inferred regulatory networks are organized into “communities” of TFs and genes collectively associated with regulation of distinct biological processes. We used a bipartite community detection method to explore the structure of the *Mtb* regulatory network and found communities of genes and TFs that display condition-specific pathway enrichment. These include an inhibitor-specific community over-represented for genes involved in ATP production; this result was validated experimentally.

Taken together, these results demonstrate gene regulatory network inference using PANDA can effectively integrate multi-omic data and infer regulatory networks that capture downstream signaling effects. This, combined with advances in high-throughput methods for measuring phosphorylation-dependent protein–protein interactions^26^, creates new opportunities for the functional characterization of drug candidates in *Mtb*. Approaches such as those described here will be essential for finding interventions in a disease that is already a substantial threat to human health and is becoming increasingly difficult to treat.

## Methods

### RNA-seq and phosphoproteomics data

RNA-Seq and phosphoproteomics data collection, processing and results are available in ref. ^6^. For the RNA-Seq used to generate the PANDA networks, there were 27 samples each for the inhibitor- and control-treated conditions (3 individual experiments x 3 replicates x 3 time points). The RNA-Seq is available through the Gene Expression Omnibus (GEO) database under the accession number GSE110508, and protein phosphorylation data is available from the ProteomeXchange Consortium via the PRIDE partner repository with the dataset identifier PXD008968.

### Reconstructing PANDA networks

The PANDA method is an approach for estimating TF regulatory networks based on our understanding of how TFs regulate genes. Specifically, it models TF regulation based on three assumptions: (1) a TF that binds the promoter region of a gene is more likely to regulate that gene, (2) pairs of TFs, such as those within the same multi-protein complex, are more likely to regulate some of the same genes, (3) pairs of genes that are correlated in their expression are more likely to be regulated by some of the same TFs. For (1), PANDA begins with an unweighted regulatory network prior of TF-gene binding interactions. For (2), PANDA uses a TF-TF cooperativity network where an edge exists if there is evidence that the two TFs interact. For (3), PANDA starts with a gene-gene co-expression network where each edge is weighted based on the Pearson correlation of the expression levels between two genes.

Using these as inputs, PANDA then calculates two functions at each timepoint *t*: (a) The *responsibility*, which estimates the support for an edge between a TF and a gene based on the similarity between the other regulators of that gene in the regulatory network and the interactions of that TF in the TF-TF cooperativity network, and (b) the *availability*, which estimates the support for an edge between a TF and gene based on the similarity between the other genes targeted by that TF in the regulatory network and the other genes that are co-regulated with that gene in the gene co-expression network. The edge is then updated based on the average of the *responsibility* and *availability*. The TF-TF cooperativity and gene-gene co-expression networks are then also updated based on the similarity of the genes targeted by each pair of TFs and the TFs targeting each pair of genes, respectively. In each of these comparisons, the similarity is calculated using the same function, which is a modified Tanimoto. This process continues until the algorithm converges, resulting in a regulatory network containing TF-gene edges whose weights represent combined support from the three input data types (Supplementary Fig. 7). For additional details, see ref. ^9^.

#### PANDA regulatory network prior

We created the regulatory network prior using ChIP-seq data from the supplemental material of ref. ^7^. The regulatory network prior contains 6517 TF binding interactions between 143 TFs and 2501 genes, filtered by significance (*P* < 0.01) and located within the −150 to +70 nucleotide promoter window. In reconstructing networks, we consider only these 143 TFs as defined in ref. ^7^ as potential regulators. The regulatory network prior is available at 10.5281/zenodo.3960874.

#### PANDA protein-cooperativity network prior

Predicted interactions between TFs were obtained from STRING v10^8^. We filtered these interactions to include only those between the 143 TFs in our regulatory network prior. PANDA accounts for the strength of transcription factor protein–protein interactions, and thus we used all TF-TF combined scores calculated by STRING as a network input. We examined the effect of thresholding the protein-cooperativity network to include only the top 25% most confident edges as well as the effect of including eight additional edges representing physical protein–protein interaction data^27^. We observed similarly high correlations of the (inhibitor-control) edge weights between the primary analysis and these sensitivity analyses, with an (inhibitor-control) edge weight Spearman correlation of 0.997 and 1.000, respectively (Supplementary Data 6). The protein cooperativity prior is available at 10.5281/zenodo.3960874.

#### Computing PANDA networks and transcription factor and gene node strength

We ran PANDA (R package pandaR version 1.4.2, downloaded from https://www.bioconductor.org/packages/release/bioc/html/pandaR.html) with the default update parameter (*α* = 0.10) using the same TF-gene regulatory network and protein–protein TF cooperativity priors, but with gene expression data unique to samples treated with the active compound or control compound; the input gene co-expression priors are available at 10.5281/zenodo.3960874. While PANDA outputs a final regulatory network, a final transcription factor protein-cooperativity network, and a final co-expression network, all downstream analyses used only the regulatory network.

We computed TF node strength as the sum of all outbound edge weights from a TF node in a PANDA network. The reason we compute TF node strength instead of standard outdegree (number of outbound edges) is because PANDA estimates edge weights for all possible TF-gene pairs. Thus, network differences are conveyed through differences in edge weights rather than which edges exist. Analogously, gene node strength is the sum of all inbound edge weights for a gene node in a PANDA network.

We also examined the effect of varying *α*, the PANDA update parameter. For each condition, we observed high correlation of edge weight differences between the primary analysis network (*α* = 0.1) and networks we created with *α* = 0.05 and *α* = 0.2, with an (inhibitor-control) edge weight Spearman correlation of 0.999 and 0.998, respectively (Supplementary Data 6).

### ATP quantification

Triplicate samples from three individual experiments were grown and harvested at three serial time points as previously described^6^. Metabolic activity was rapidly quenched by placing the bacteria directly into 40% acetonitrile, 40% methanol, 20% water previously cooled on dry ice. The cells were then mechanically disrupted with 0.1 mm Zirconia beads in a MagNA Lyser instrument (Roche) by agitating the samples four times at 7000 rpm for 45 s with a cooling step at −20 ^∘^C for 5 min between each cycle. The lysate was then clarified by centrifugation (10,000 × *g*, 10 min, 4^ ∘^C) and filtered through a 0.22 μm filter (Costar^®^ #8160). ATP quantification was performed according to the manufacturer’s instructions of the ATP Colorimetric/Fluorometric Assay kit (BioVision #K354-100) and normalized to the residual protein quantity, determined by the Pierce^TM^ BCA Protein Assay kit (ThermoFisher #23227).

### Functional annotations

Gene Ontology (GO) and KEGG Pathway functional categories were downloaded from PATRIC^28^. We removed duplications and functional categories matching the regular expression eukary∣plant∣(?i)photosynth∣E. Coli∣bile∣insect. To these we added manually curated functional categories (Supplementary Data 7).

### Statistical significance of PknA/PknB-specific TFs and genes

We used permutation testing to calculate empirical *P*-values for the significance of differential targeting for each TF and gene. To do this, we ran PANDA for each of 1000 randomized gene expression matrices with permuted gene labels. For each TF/gene, we then calculated significance by determining the proportion of the node strength differences for the 1000 runs that were greater than the observed node strength if positive, or less than the observed node strength if negative. The minimum possible *P*-value attainable by permutation testing was 0.001, and we were limited from obtaining more precise *P*-values given computational expense. We additionally computed p-values for TF/gene node strength by performing a paired two-sided *t*-test of all edge weights associated with a TF/gene for the inhibitor vs. control condition. We performed Benjamini–Hochberg multiple testing correction for these *P*-values computed using the *t*-test.

### Bipartite network community detection

We used the CONDOR R package^25^ with project = FALSE and other parameters set to default to detect communities in the inhibitor and control networks separately. These two networks contained the same subset of edges, but had different edge weights based on the output from running PANDA with data from the two conditions. Edges with weight < 0 in either network were removed from both networks.

### Functional enrichment analysis

To identify PknA/PknB-specific functional categories, we ran GSEA Preranked, which we downloaded from https://www.broadinstitute.org/gsea/as the Java version 2.0.13. We ranked TFs/genes by their difference (inhibitor − control) in TF/gene node strength and ran GSEA using a minimum gene set size of 10 and a maximum size of 250. We report statistically significant results (FDR < 0.1), with positive and negative enrichment scores representing enrichment in the inhibitor and control treatments, respectively. We used the one-sided Fisher’s Exact Test to evaluate the significance of each functional category for a given gene set. We required a minimum overlap of five genes between the gene set and the genes annotated to the functional category for significance to be considered. Multiple testing correction was done using the Benjamini–Hochberg method.

## Supplementary information

Supplementary Data 1

Supplementary Data 2

Supplementary Data 3

Supplementary Data 4

Supplementary Data 5

Supplementary Data 6

Supplementary Data 7

Supplementary Information

## Data Availability

Data to reproduce the results can be found at 10.5281/zenodo.3960874. The RNA-Seq used for this work is available through the Gene Expression Omnibus (GEO) database under the accession number GSE110508, and protein phosphorylation data is available from the ProteomeXchange Consortium via the PRIDE partner repository with the dataset identifier PXD008968.

## Code availability

Code to reproduce the results can be found at 10.5281/zenodo.3960874.

## References

[1] World Health Organization. Tuberculosis URL https://www.who.int/news-room/fact-sheets/detail/tuberculosis (2020).

[2] Russell DG (2010). *Mycobacterium tuberculosis* wears what it eats. Cell Host Microbe.

[3] Molle V, Kremer L (2010). Division and cell envelope regulation by Ser/Thr phosphorylation: Mycobacterium shows the way. Mol. Microbiol..

[4] Ruggiero A, De Simone P, Smaldone G, Squeglia F, Berisio R (2012). Bacterial cell division regulation by Ser/Thr kinases: a structural perspective. Curr. Protein Pept. Sci..

[5] Kang C-M (2005). The *Mycobacterium tuberculosis* serine/threonine kinases PknA and PknB: substrate identification and regulation of cell shape. Genes Dev..

[6] Carette X (2018). Multisystem analysis of *Mycobacterium tuberculosis* reveals kinasedependent remodeling of the pathogen-environment interface. mBio.

[7] Minch KJ (2015). The DNA-binding network of *Mycobacterium tuberculosis*. Nat. Commun..

[8] Szklarczyk D (2015). STRING v10: protein-protein interaction networks, integrated over the tree of life. Nucleic Acids Res..

[9] Glass K, Huttenhower C, Quackenbush J, Yuan G-C (2013). Passing messages between biological networks to refine predicted interactions. PLoS ONE.

[10] Galagan JE (2013). The *Mycobacterium tuberculosis* regulatory network and hypoxia. Nature.

[11] Haydel SE, Clark-Curtiss JE (2006). The *Mycobacterium tuberculosis* TrcR response regulator represses transcription of the intracellularly expressed Rv1057 gene, encoding a seven-bladed beta-propeller. J. Bacteriol..

[12] Blasco B (2012). Virulence regulator EspR of *Mycobacterium tuberculosis* is a nucleoidassociated protein. PLoS Pathog..

[13] Bartek IL (2014). *Mycobacterium tuberculosis* Lsr2 is a global transcriptional regulator required for adaptation to changing oxygen levels and virulence. mBio.

[14] Liu T (2007). CsoR is a novel *Mycobacterium tuberculosis* copper-sensing transcriptional regulator. Nat. Chem. Biol..

[15] Ward SK, Hoye EA, Talaat AM (2008). The global responses of *Mycobacterium tuberculosis* to physiological levels of copper. J. Bacteriol..

[16] Kendall SL (2010). Cholesterol utilization in mycobacteria is controlled by two TetRtype transcriptional regulators: kstR and kstR2. Microbiology (Reading, England).

[17] Sala A, Bordes P, Genevaux P (2014). Multiple toxin-antitoxin systems in *Mycobacterium tuberculosis*. Toxins.

[18] Pinto R, Tang QX, Britton WJ, Leyh TS, Triccas JA (2004). The *Mycobacterium tuberculosis* cysD and cysNC genes form a stress-induced operon that encodes a tri-functional sulfate-activating complex. Microbiology (Reading, England).

[19] Gordhan BG (2002). Construction and phenotypic characterization of an auxotrophic mutant of *Mycobacterium tuberculosis* defective in L-arginine biosynthesis. Infect. Immun..

[20] Tiwari S (2018). Arginine-deprivation-induced oxidative damage sterilizes *Mycobacterium tuberculosis*. Proc. Natl Acad. Sci. USA.

[21] De Voss JJ (2000). The salicylate-derived mycobactin siderophores of *Mycobacterium tuberculosis* are essential for growth in macrophages. Proc. Natl Acad. Sci. USA.

[22] Portevin D (2004). A polyketide synthase catalyzes the last condensation step of mycolic acid biosynthesis in mycobacteria and related organisms. Proc. Natl Acad. Sci. USA.

[23] McMahon MD, Rush JS, Thomas MG (2012). Analyses of MbtB, MbtE, and MbtF suggest revisions to the mycobactin biosynthesis pathway in *Mycobacterium tuberculosis*. J. Bacteriol..

[24] McLaughlin B (2007). A Mycobacterium ESX-1 secreted virulence factor with unique requirements for export. PLoS Pathogen..

[25] Platig J, Castaldi PJ, DeMeo D, Quackenbush J (2016). Bipartite community structure of eQTLs. PLoS Comput. Biol..

[26] Barber KW (2018). Encoding human serine phosphopeptides in bacteria for proteome-wide identification of phosphorylation-dependent interactions. Nat. Biotechnol..

[27] Wang Y (2010). Global protein protein interaction network in the human pathogen *Mycobacterium tuberculosis* H37Rv. J. Proteome Res..

[28] Wattam AR (2014). PATRIC, the bacterial bioinformatics database and analysis resource. Nucleic Acids Res..

[29] Barber MJ (2007). Modularity and community detection in bipartite networks. Phys. Rev. E.

