# Supplementary material for: Multi-omic regulatory networks capture downstream effects of kinase inhibition in Mycobacterium tuberculosis
Source: NPJ Syst Biol Appl. 2021 Jan 29;7:8. doi: 10.1038/s41540-020-00164-4 (PMC7846781; doi:10.1038/s41540-020-00164-4)
Supplement: Supplementary file 8 — Supplementary Information [file 41540_2020_164_MOESM8_ESM.pdf]

Supplementary Information

Supplementary Figures.

- **Supplementary Figure 1.** Comparison of edge weights between the inhibitor and control PANDA networks
- **Supplementary Figure 2.** The differential expression of a gene is related to its change in node strength (being differentially targeted)
- **Supplementary Figure 3.** Node strength distributions of thresholded PANDA networks
- **Supplementary Figure 4.** Comparison of community structure between inhibitor and control networks
- **Supplementary Figure 5.** Comparison of community overlaps between conditions
- **Supplementary Figure 6.** The effect of threshold used to define PANDA subnetworks for CONDOR community detection
- **Supplementary Figure 7.** Overview of the PANDA algorithm for TF network reconstruction

Supplementary Data Descriptions.

Supplementary References.

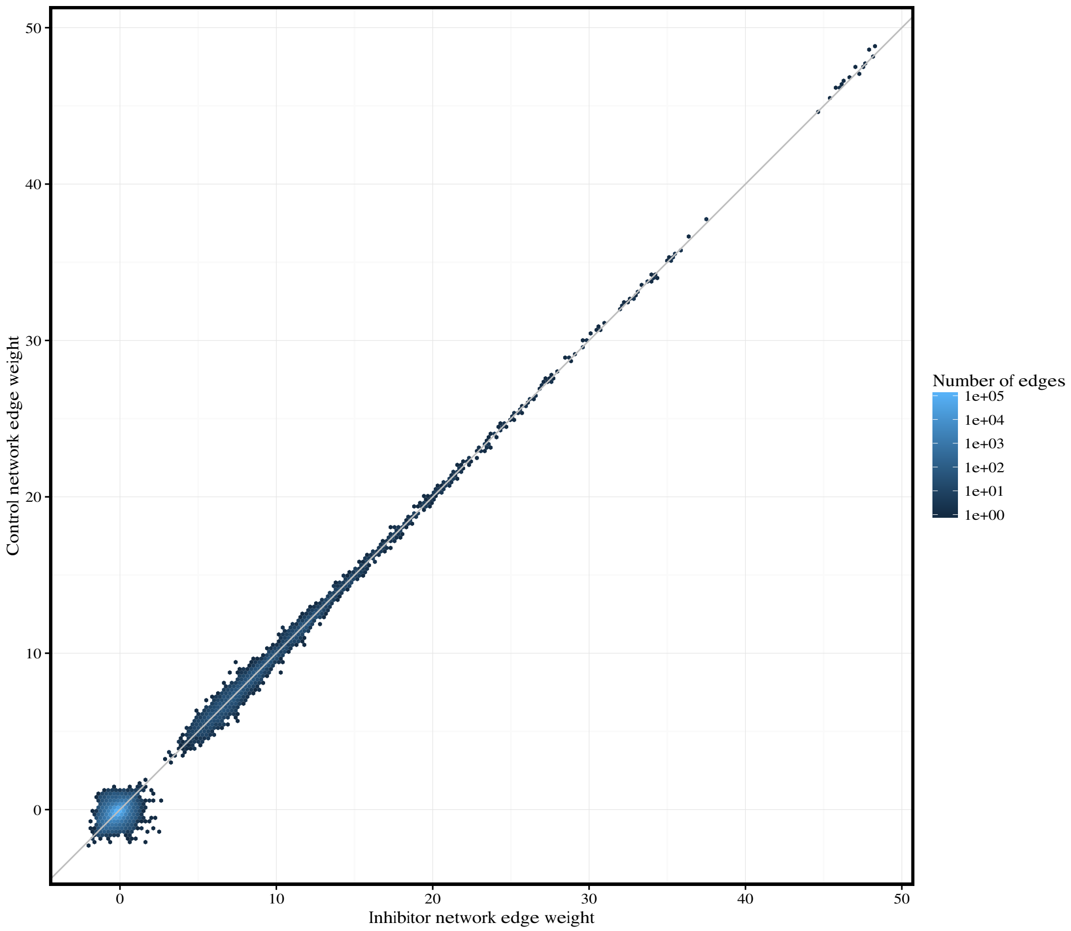

Supplementary Figure 1. Comparison of edge weights between the inhibitor and control PANDA networks. Edge weights are in z-score units.

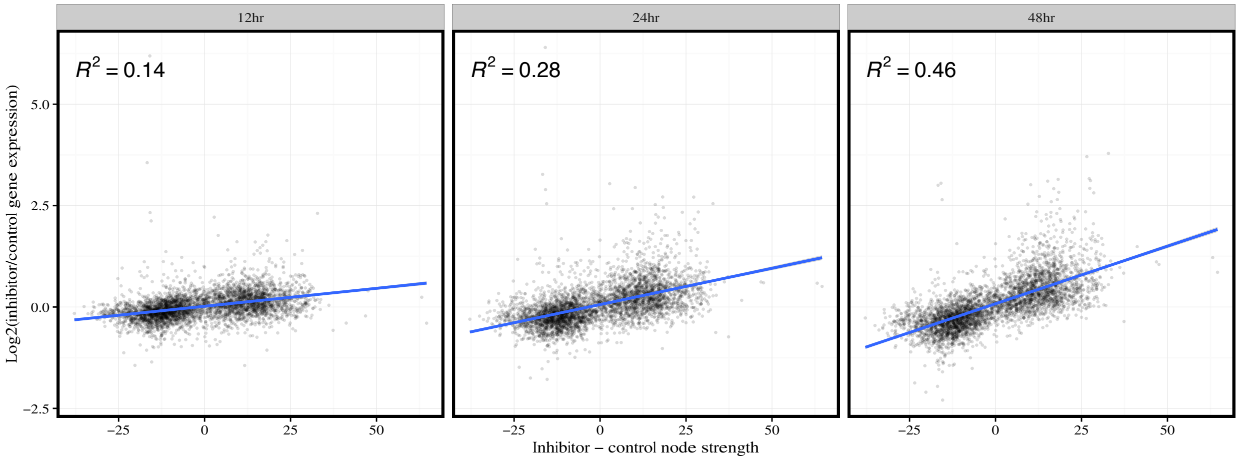

Supplementary Figure 2. The differential expression of a gene is related to its change in node strength (being differentially targeted). Plots are shown for differential expression at each experimental time point (12 hours, 24 hours, and 48 hours).[1] The best fit line is shown for each plot. The coefficient of determination is shown in text.

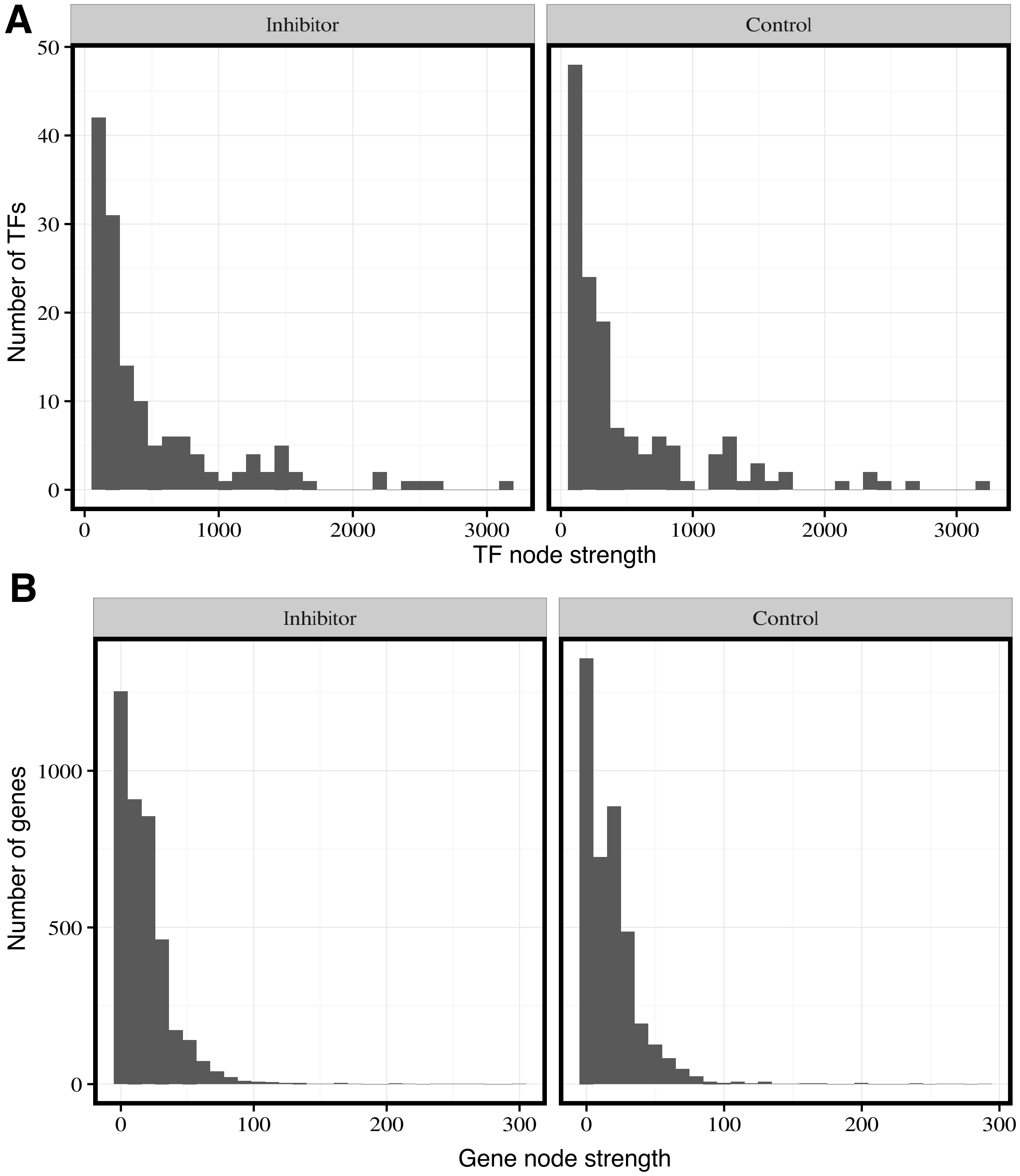

Supplementary Figure 3. Node strength distributions of thresholded PANDA networks. (A) Histogram of the node strength per transcription factor in each condition. (B) Histogram of the node strength per gene in each condition.

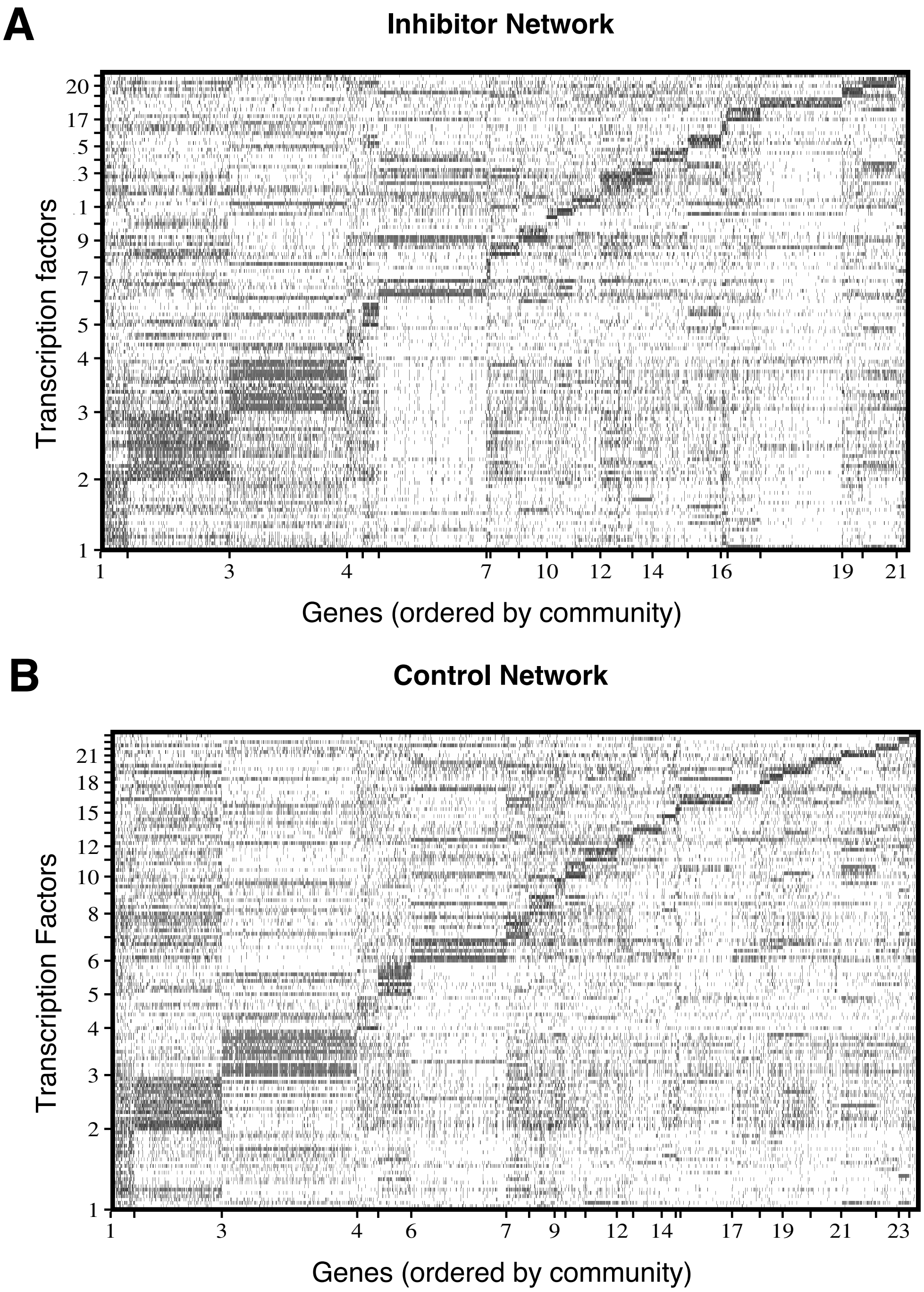

Supplementary Figure 4. Comparison of community structure between inhibitor and control networks. (A) Heatmap of edges between the 143 transcription factors and 3971 genes for the thresholded inhibitor PANDA network, sorted by community membership. Shaded regions represent the existence of an edge with weight greater than 0. Transcription factors and genes are arranged along rows and columns, respectively. The axis labels depict the community number. (B) Corresponding heatmap for control PANDA network.

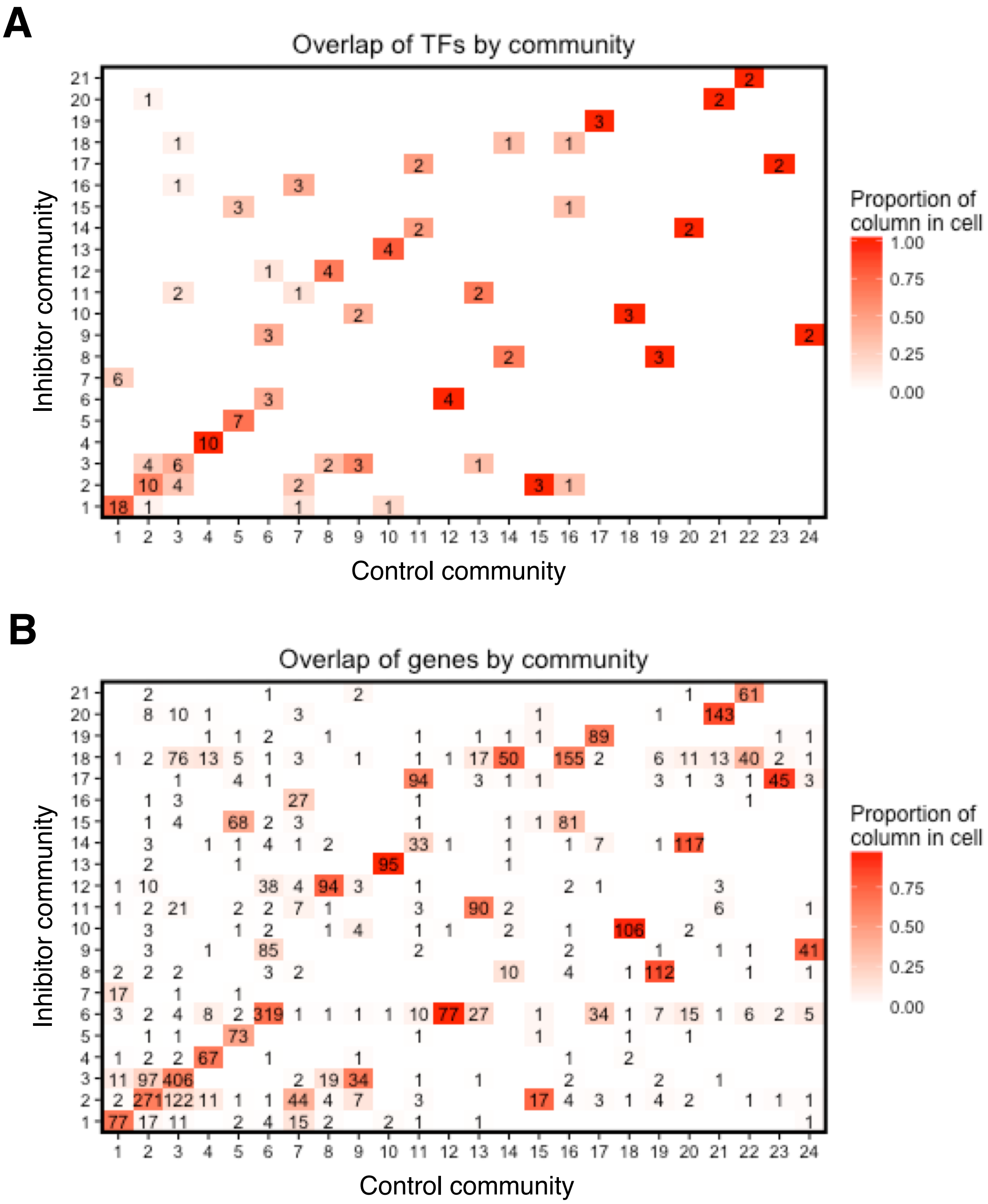

Supplementary Figure 5. Comparison of community overlaps between conditions. The value in each cell is the number of (A) TFs or (B) genes assigned to both the corresponding inhibitor-treated and control-treated communities. The red shading helps to visualize how the TFs/genes in each control community are distributed among inhibitor communities.

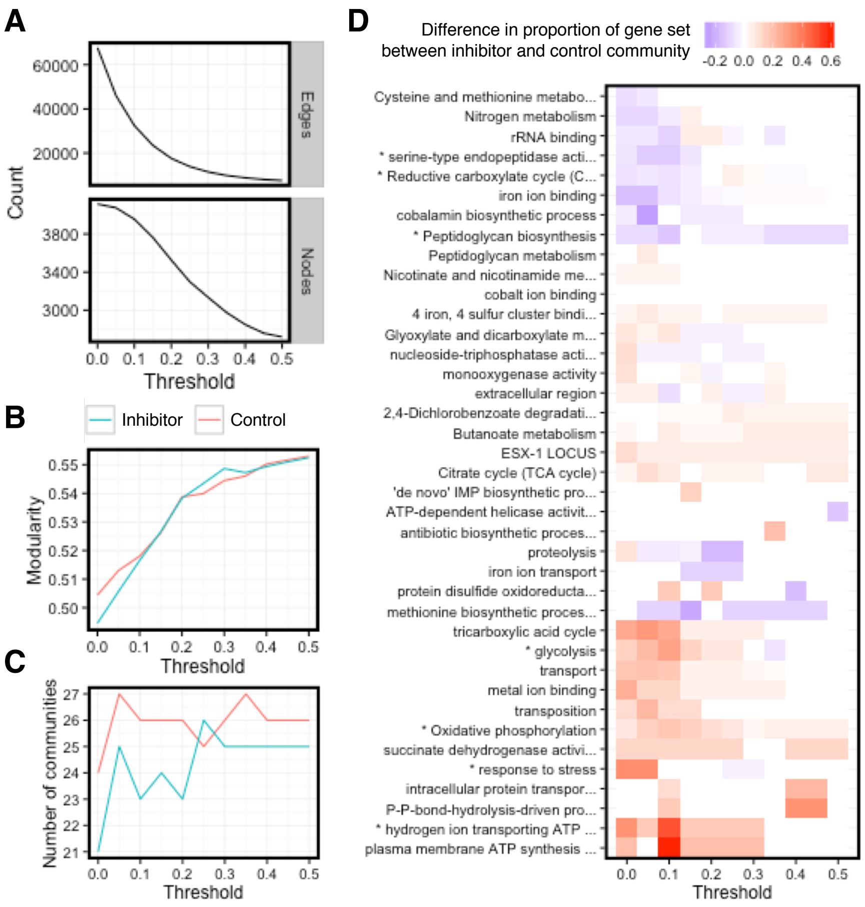

Supplementary Figure 6. The effect of threshold used to define PANDA subnetworks for CONDOR community detection. (A) Number of edges that would be assigned to each subnetwork depending on the threshold (z-score cutoff) used. (B) Modularity of the network clustering per subnetwork depending on threshold. (C) Number of communities detected depending on threshold. (D) We select functional categories that show significant enrichment in at least 5 thresholds in either condition, together with functional categories that were significant at a threshold of 0. At each threshold and for each condition, we compute the proportion of genes in the network in the functional category present in the community containing the highest proportion of genes in the functional category. We then take the difference in this proportion between communities. For example, an inhibitor - control proportion of 0.5 may suggest that at a particular threshold, 0.75 of the genes in the functional category were present in the same community in the inhibitor, while only 0.25 of the genes in the functional category were present in the same community in the control network.

We plot this difference in proportion for each functional category and at each threshold. Functional categories deemed significant at a threshold of 0 and discussed in the text are marked with an asterisk (*).

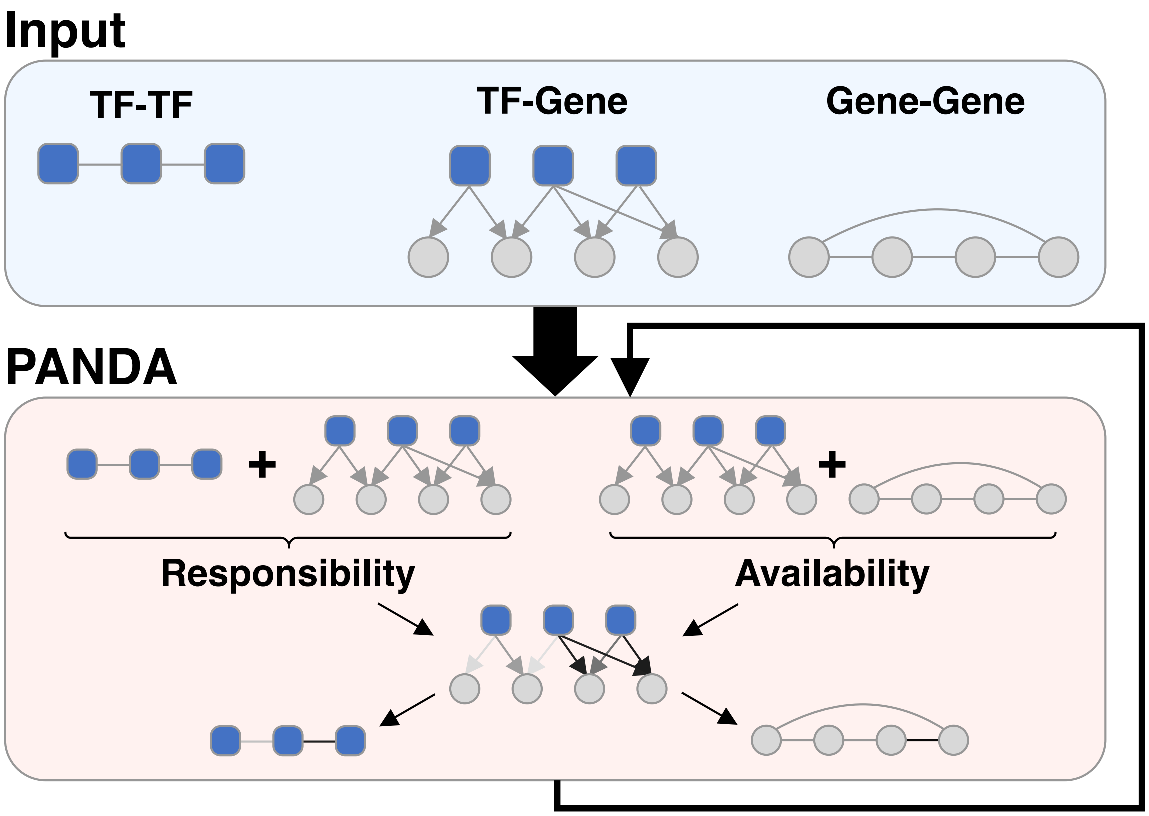

Supplementary Figure 7. Overview of the PANDA algorithm for TF network reconstruction. PANDA takes as input three networks: a TF-TF cooperativity network, a TF-gene prior network and a gene-gene co-expression network. For each iteration, the method uses message passing to update the TF-gene edges, then updates the TF-TF and gene-gene edges. This process repeats until convergence.

Supplementary Data 1. Differential targeting and differential expression of network transcription factors.

Supplementary Data 2. Differential targeting and differential expression of network genes.

Supplementary Data 3. Differential targeting of functional categories identified by gene set enrichment analysis (GSEA).

Supplementary Data 4. Overrepresented functional categories in inhibitor communities identified by the one-sided Fisher's Exact Test.

Supplementary Data 5. Overrepresented functional categories in control communities identified by the one-sided Fisher's Exact Test.

Supplementary Data 6. Sensitivity analysis results: Spearman correlations between new (sensitivity analysis) and original (primary analysis) networks using (1) differences (inhibitor - control) in edge weight, (2) inhibitor edge weights, and (3) control edge weights to compare different network reconstructions.

Supplementary Data 7. Curated functional categories.

Supplementary References

1. Carette, X. *et al.* Multisystem Analysis of Mycobacterium tuberculosis Reveals Kinase-Dependent Remodeling of the Pathogen-Environment Interface. *MBio* **9**, e02333-17 (2018).
